# RBAtools: a programming interface for Resource Balance Analysis models

**DOI:** 10.1093/bioadv/vbad056

**Published:** 2023-04-22

**Authors:** Oliver Bodeit, Inès Ben Samir, Jonathan R Karr, Anne Goelzer, Wolfram Liebermeister

**Affiliations:** MaIAGE, Université Paris-Saclay, INRAE, 78350 Jouy-en-Josas, France; Institute of Quantitative and Theoretical Biology, Heinrich-Heine-Universität Düsseldorf, 40225 Düsseldorf, Germany; Institute of Biology, Theoretical Biophysics, Humboldt-Universität zu Berlin, 10115 Berlin, Germany; Institute of Biochemistry, Charité—Universitätsmedizin Berlin, 10117 Berlin, Germany; MaIAGE, Université Paris-Saclay, INRAE, 78350 Jouy-en-Josas, France; Department of Genetics and Genomic Sciences, Icahn School of Medicine at Mount Sinai, New York, NY 10029, USA; MaIAGE, Université Paris-Saclay, INRAE, 78350 Jouy-en-Josas, France; MaIAGE, Université Paris-Saclay, INRAE, 78350 Jouy-en-Josas, France

## Abstract

**Motivation:**

Efficient resource allocation can contribute to an organism’s fitness and can improve evolutionary success. Resource Balance Analysis (RBA) is a computational framework that models an organism’s growth-optimal proteome configurations in various environments. RBA software enables the construction of RBA models on genome scale and the calculation of medium-specific, growth-optimal cell states including metabolic fluxes and the abundance of macromolecular machines. However, existing software lacks a simple programming interface for non-expert users, easy to use and interoperable with other software.

**Results:**

The python package RBAtools provides convenient access to RBA models. As a flexible programming interface, it enables the implementation of custom workflows and the modification of existing genome-scale RBA models. Its high-level functions comprise simulation, model fitting, parameter screens, sensitivity analysis, variability analysis and the construction of Pareto fronts. Models and data are represented as structured tables and can be exported to common data formats for fluxomics and proteomics visualization.

**Availability and implementation:**

RBAtools documentation, installation instructions and tutorials are available at https://sysbioinra.github.io/rbatools/. General information about RBA and related software can be found at rba.inrae.fr.

## 1 Introduction

How can we understand and anticipate the impact of genomic modifications and environmental perturbations on microbial cells? A guiding idea is that organisms efficiently allocate their resources to succeed within their ecological niche (Goelzer *et al.*, 2011; Molenaar *et al.*, 2009; Scott *et al.*, 2010). Resource Balance Analysis (RBA) is a conceptual and computational framework that implements this principle as a constraint-based modelling method (Varma and Palsson, 1994) predicting growth-optimal cell states (Goelzer *et al.*, 2011, 2015). RBA can simulate responses to genomic (e.g. gene-knockouts and the addition of heterologous metabolic pathways) and environmental perturbations (e.g. nutrient limitation). Due to its formulation as a linear optimization problem (LP), it can handle cell models at genome scale.

**Fig. 1.**
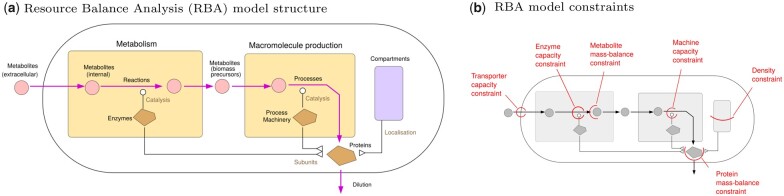
Resource Balance Analysis models. (**a**) Model structure. An RBA model describes production and consumption processes in an entire cell, catalysed by molecular machines. Fluxes and machine concentrations must be chosen to realize a balanced growth state in which all cell components are reproduced to balance dilution. The cell configuration may be optimized for maximal growth or for maximizing a side objective at a given, sub-maximal growth rate. Unlike Flux Balance Analysis (FBA), RBA contains a detailed description of anabolic processes such as protein synthesis, folding and transport, and instead of assuming a predefined biomass composition, it returns an optimal cell composition as a result. Background information on RBA, including literature references and more details about model structure, parameters and file formats, can be found at rba.inrae.fr. (**b**) In RBA, the model variables (metabolic fluxes, amounts of molecular machines and maybe other variables) must respect four types of constraints: mass balance constraints ensuring a balanced growth state; catalytic capacity constraints relating fluxes to catalyst concentrations; density constraints limiting the compound concentrations in cells; and target constraints representing empirical knowledge, for instance about concentrations of macromolecules without a specified function. Most constraints come with individual parameters such as catalyst efficiencies (apparent $k_{\text{cat}}$ values) of enzymes and process machines, the available space in cell compartments, and empirical concentrations and fluxes for target constraints

The basic structure of RBA models is shown in Figure 1. An RBA model extends a genome-scale metabolic model and describes production processes and molecular machines in a cell (metabolic enzymes and machines catalysing macromolecular processes such as protein translation, folding or transport). It may contain hundreds or thousands of model parameters, specifying enzyme and machine efficiencies, compartment volumes and known target concentrations. Mathematically, the search for feasible cell states takes place at a given cell growth rate. At high growth rates, the problem becomes unsolvable, thus defining a maximal feasible grow rate and a corresponding optimal state. Typical simulation scenarios include maximizing growth or optimizing metabolic objectives at a given sub-maximal growth rate, for instance, the production of valuable compounds.

The existing software RBApy (Bulović *et al.*, 2019) supports the construction of genome-scale RBA models as well as the basic prediction of maximal growth rates and corresponding cellular states. It has been used to develop RBA models for several bacterial organisms (Bulović *et al.*, 2019; Goelzer *et al.*, 2015), encoded in a standard XML format. However, RBApy lacks functionality for convenient model editing, for exploring resource allocation beyond growth-optimal states, and for implementing custom workflows for model simulation and analysis.

## 2 RBAtools

RBAtools is a programming interface for RBA models that covers some functionality of RBApy and adds functions for exploring and modifying a model or for performing different types of simulations and analyses. RBAtools provides basic functions for setting parameters or manipulating and solving the LP problem, as well as convenient methods for altering model architectures and defining growth environments. Basic simulations based on growth-rate maximization can be run programmatically or via the command line. RBAtools also offers high-level functions for typical simulation and analysis tasks occurring in resource allocation modelling. Based on these built-in procedures, users can implement their own custom algorithms and workflows for simulation and analysis. RBAtools gives programmatic access to model components and their relationships and can export models and simulation results into formats such as SBtab (Lubitz *et al.*, 2016) or CSV. For visualizing predicted fluxes and protein levels, it can export data files for Escher maps (King *et al.*, 2015) and Proteomaps (Liebermeister *et al.*, 2014). Instructive tutorials and a *Bacillus subtilis* cell model are included. Below we present example results, showcasing some high-level functions for model simulation and analysis from the RBAtools tutorial.

### 2.1 Prediction of cell phenotypes for biotechnology

**Fig. 2.**
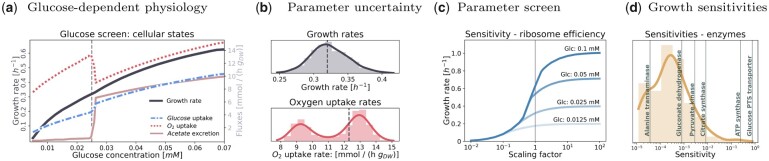
Cell phenotypes and parameter sensitivities computed with RBAtools. The plots show results for the genome-scale *B.subtilis* cell model, taken from the RBAtools tutorial. (**a**) Monod curve depicting the glucose-dependent cell growth rate. In the model with glucose as the only carbon source, Monod curve and corresponding exchange fluxes of glucose, oxygen and acetate were computed by screening the external glucose concentration and optimizing for maximal growth. At a glucose concentration of 0.025 mM, cells switch from respiration to overflow metabolism. This concentration serves as a reference condition for subsequent analyses (dashed black lines). (**b**) Global sensitivity analysis. The plots show the variability of maximal growth rates and associated oxygen uptake rates in a model ensemble with enzyme efficiencies sampled at random. Each enzyme efficiency in the model is multiplied by a random scaling factor *x*, where ln(x) is drawn from a normal distribution with mean 0 and standard deviation ln(1.1). The histograms of maximal growth rates and optimal oxygen uptake rates represent an ensemble of 1000 models. In the distribution of oxygen uptake rates, the two modes correspond to respiration and overflow metabolism, metabolic strategies that the cells would respectively use at lower or higher glucose concentrations. At the reference concentration of 0.25 mM (critical concentration in the model with standard parameters), the two strategies are equally profitable and small variations of enzyme efficiencies can be a scale-tipping factor. (**c**) Parameter screen. The predicted optimal growth rate (*y*-axis) depends on ribosome efficiency (*x*-axis) and on the external glucose concentration. Curves for different glucose concentrations are shown. Ribosomal efficiencies were screened by applying a scaling factor between 0.01 and 100. (**d**) Local parameter sensitivities. In the optimal state, active enzymes (with enzyme efficiency $k_{\text{app}}$) have a positive control over the maximum growth rate $\mu^{\text{max}}$, where control is quantified by unitless scaled parameter sensitivities $d\ln(\mu^{\text{max}})/d\ln(k_{\text{app}})$. Among the 587 metabolic enzymes, 245 enzymes show non-zero sensitivities (shown as a histogram, some prominent enzymes are marked by lines). The growth/efficiency sensitivities of enzymes and transporters located in membranes are especially high

RBAtools provides various modelling methods for biotechnology, for example to simulate the production of added-value compounds. Models can be edited to simulate gene knock-outs, enzyme inhibition, enzyme overexpression or underexpression, changes in cell dry-mass composition or the insertion of heterologous metabolic pathways. Known cell characteristics such as metabolite exchange fluxes or macromolecular machine abundances can be imposed as target constraints and the resulting phenotype (maximum growth rate, metabolic fluxes and quantitative proteome) can be predicted. It is possible to compute Monod curves and the associated medium exchange rates (Fig. 2a) and to infer the minimum concentration of a limiting substrate at which given growth rate can be reached (to model, for example, chemostat experiments).

### 2.2 Sensitivity to model parameters

To study the effects of parameter variation and uncertainty, RBAtools provides different types of sensitivity analyses. (i) In a global variability analysis, the effects of variable or uncertain enzyme efficiencies on fluxes or other state variables can be assessed by random sampling (Fig. 2b). (ii) By screening single parameter values and predicting cellular states, effects on the phenotype can be assessed, as exemplified by the ribosomal translation efficiency curve in Figure 2c. (iii) Local growth sensitivities, defined as scaled derivatives between growth rate and a parameter value, can be computed for all model parameters (sensitivities to enzyme efficiencies shown in Fig. 2d).

### 2.3 Variability analysis and cellular trade-offs

Bioengineering has to deal with trade-offs between compound production and a cell’s own objectives such as cell growth or the capacity to respond to stress. Compound production redirects resources such as energy, precursors or available cell space towards non-native processes, which puts a burden on cells and slows down cell growth. In engineered microbes, slower growth can lead to the emergence of non-producing, fast-growing mutants. To anticipate and avoid this, we may simulate trade-offs between growth and objectives such as protein or ATP production. In RBA, growth-optimal states usually do not leave any space for variations, so there is no extra capacity for other processes. At sub-maximal growth rates, cells can deviate from the growth-optimal phenotype, and since this tolerance tends to increase with decreasing cell growth, there can be a trade-off between growth and other objectives. RBAtools provides methods to study such trade-offs.

**Fig. 3.**
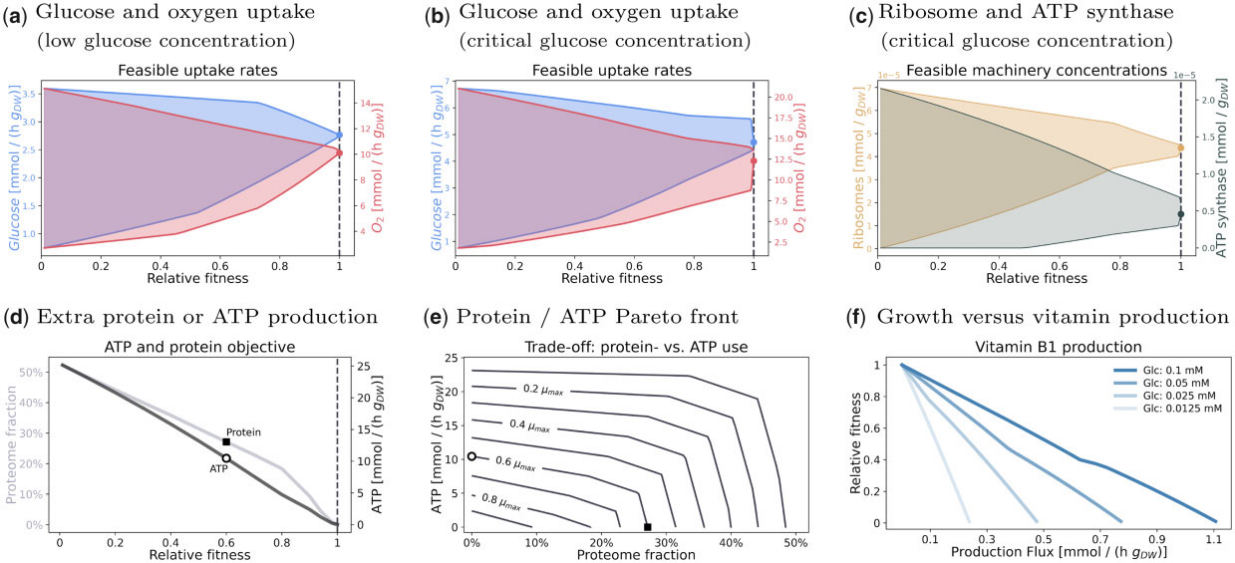
Cellular trade-offs in the *B. subtilis* model analysed with RBAtools. (**a**) Resource Variability Analysis (RVA). Feasible ranges of glucose and oxygen uptake (*y*-axis) depend on cell growth (*x*-axis shows growth rates normalized to maximal growth as ‘relative fitness’). Above the maximal growth rate (dashed line) there is no solution. At the maximum growth rate, the feasible region collapses to a single optimal point (dots). Panel (a) shows results at low glucose (0.0125 mM), where respiration takes place. (**b**) RVA results at medium glucose concentration (0.025 mM, the onset of overflow metabolism): here respiration and overflow are equally profitable, the cell configuration is highly flexible, and even slightly sub-maximal growth rates allow for large feasible ranges. (**c**) RVA of machine amounts (ribosome and ATP synthase) at medium glucose concentration (onset of overflow). Ribosome ranges increase at lower growth rates, indicating a trade-off between ribosome amount and cell growth. (**d**) Trade-off between growth rate and two metabolic objectives. At sub-maximal growth, cells may allocate resources to additional ATP expenditure or non-native protein expression. Figure (d) depicts the trade-off between relative growth rate and extra protein production (as a fraction of the total proteome) or an extra ATP-turnover flux. The curves are upper edges of feasible regions as shown in (a–c). Points marked by symbols correspond to extremal Pareto-efficient trade-offs shown in panel (**e**). (e) Pareto-efficient trade-offs between investments in non-native cytosolic protein or in ATP turnover at various fractions of the maximal growth rate ($\mu_{\text{max}}$). Lines represent Pareto fronts at different growth rates. Marked points correspond to the points in (d). (**f**) Trade-off between cellular fitness (growth rate as a fraction of maximal growth rate) and metabolite production in a biotechnological application. The maximum production of vitamin B1 is plotted against cell fitness at different glucose concentrations in the growth medium

Resource Variability Analysis (RVA) resembles Flux Variability Analysis (Mahadevan and Schilling, 2003), but is applied to RBA models. It assumes a predefined, sub-maximal growth rate and determines feasible ranges of metabolic fluxes and machinery concentrations. A large tolerance range around a growth-optimal phenotype can indicate biological variability (at almost no growth deficit) or prediction uncertainties. If we plot these ranges as a function of growth rate (Fig. 3a), trade-offs between growth and production/consumption capabilities become visible. The feasible range of a cell variable is bounded from above and below by two converging curves, and if our variable is a maximisation objective, the upper curve can be seen as a Pareto front. Figure 3a shows a typical case: near the maximal growth rate, the two fronts converge in one point, which marks the optimal state. Below this point, there is a trade-off: higher tolerances require slower cell growth. However, the picture can change if we change the external glucose concentration. At the critical glucose concentration of 0.25 mM, respiration and overflow become equally beneficial (Fig. 3b) and a wide range of mixed strategies (with different glucose and oxygen uptake rates) can exist very close to the maximal growth rate. Even a tiny side benefit (e.g. for lower glucose uptake) would suffice to move the cell state from the exact growth maximum to the kink of the Pareto front. Figure 3c, similarly, shows the feasible ranges of two machine concentrations at the critical glucose concentration: while the fronts for the ribosome concentration almost converge, the front for ATP synthase shows a visible kink.

Plotting the compromise between growth and an extra metabolic capacity (for product production, energy production or stress response) as a Pareto front resembles phenotypic phase-plane analysis in FBA (Edwards *et al.*, 2002). But instead of trading metabolic objectives against growth, we can also define a sub-maximal growth rate and consider trade-offs between different metabolic tasks. The following panels in Figure 3 show this for an ATP-consuming maintenance reaction and for the expression of additional proteins (chaperones or pre-emptively expressed metabolic enzymes). Each of the two capacities can be traded individually against growth (Fig. 3d), but they can also be traded against each (Fig. 3e). At high growth rates, the resulting Pareto front resembles a straight line, indicating growth-neutral exchanges between the objectives at a constant ‘price’. At low growth rates, where large extra capacity is available, the front becomes strongly curved, showing a knee in which both objectives are close to their maximal values, possible indicating an optimal compromise.

Figure 3f shows the growth deficit caused by producing a compound of interest, vitamin B1, and how it depends on glucose concentrations in the medium. When plotting relative growth as ‘fitness’ against vitamin B1 secretion, we obtain fronts that are almost straight lines: vitamin production can be traded for cell growth at an almost constant, glucose-dependent ‘price’.

## 3 Conclusion

RBAtools is a convenient programming interface for RBA models, enabling a deep exploration of cell behaviour on genome scale. It is more flexible and user-friendly than existing RBA tools, leveraging the idea of cellular resource allocation to model cell physiology by combining biochemical facts, optimality considerations and organism-specific empirical knowledge. While RBApy remains the main tool for building RBA models, RBAtools with its simpler interface and broader functionality makes it easy to simulate the impact of genomic or environmental perturbations on cell phenotypes. Thereby, it supports a wide range of applications in synthetic biology, metabolic engineering or white biotechnology.

## Data Availability

The data underlying this article are available in the the git repository https://sysbioinra.github.io/rbatools/.
